# Histidine Decarboxylase Deficiency Prevents Autoimmune Diabetes in NOD Mice

**DOI:** 10.1155/2015/965056

**Published:** 2015-05-18

**Authors:** Manal Alkan, François Machavoine, Rachel Rignault, Julie Dam, Michel Dy, Nathalie Thieblemont

**Affiliations:** ^1^Université Paris Descartes, 75014 Paris, France; ^2^CNRS UMR 8147, Hôpital Necker, 75015 Paris, France; ^3^CNRS UMR 8104, Cochin Institute, 75014 Paris, France; ^4^INSERM U1016, Cochin Institute, 75014 Paris, France; ^5^Center of Excellence, LABEX Inflamex, 75014 Paris, France

## Abstract

Recent evidence has highlighted the role of histamine in inflammation. Since this monoamine has also been strongly implicated in the pathogenesis of type-1 diabetes, we assessed its effect in the nonobese diabetic (NOD) mouse model. To this end, we used mice (inactivated) knocked out for the gene encoding histidine decarboxylase, the unique histamine-forming enzyme, backcrossed on a NOD genetic background. We found that the lack of endogenous histamine in NOD HDC^−/−^ mice decreased the incidence of diabetes in relation to their wild-type counterpart. Whereas the proportion of regulatory T and myeloid-derived suppressive cells was similar in both strains, histamine deficiency was associated with increased levels of immature macrophages, as compared with wild-type NOD mice. Concerning the cytokine pattern, we found a decrease in circulating IL-12 and IFN-*γ* in HDC^−/−^ mice, while IL-6 or leptin remained unchanged, suggesting that histamine primarily modulates the inflammatory environment. Paradoxically, exogenous histamine given to NOD HDC^−/−^ mice provided also protection against T1D. Our study supports the notion that histamine is involved in the pathogenesis of diabetes, thus providing additional evidence for its role in the regulation of the immune response.

## 1. Introduction

Evidence has emerged that inflammatory mechanisms contribute to the onset and progression of type-1 diabetes (T1D). Recent data further suggest that the initiation of autoimmunity is preceded by inflammation reflected by a proinflammatory metabolic serum profile. Histamine is one of the classical inflammatory mediators. Many autoimmune diseases are associated with increased histamine levels in serum and tissue fluids, such as rheumatoid arthritis, Sjögren's syndrome, multiple sclerosis, and diabetes mellitus [1–4]. Similarly, it has been known for many years that histamine is elevated in tissue biopsies and secretions in the intestine of patients with Crohn's disease and ulcerative colitis [5, 6].

During inflammatory responses, histamine, a hydrophilic vasoactive amine, increases capillary permeability and facilitates the immune response by enhancing leukocyte rolling, adhesion, and vascular extravasation of inflammatory cells to the site of inflammation. Histamine is recognized not only as an inflammatory mediator but also as a regulator of immune responses, including the Th1/Th2 balance and hematopoiesis [7]. Patients with diabetes are more susceptible to vascular diseases, which led to the assumption that their increased circulating histamine levels promoted lipolysis and facilitated the transport of lipids into tissues. Beside its physiological role in vascular health, excess histamine, a result of physical or emotional stress or a chronic disease/inflammatory state, appears to elicit atherogenic effects [8]. Whether histamine is a critical marker or target in the treatment of type-1 diabetes has not yet been established.

It is still poorly understood how the expression of histidine decarboxylase (HDC), the unique enzyme responsible for histamine generation, is controlled. HDC is induced in a variety of tissues in response to bacterial components (lipopolysaccharides, peptidoglycan) and to various cytokines such as IL-1, IL-3, IL-12, TNF, G-CSF, and GM-CSF. Its upregulation has been associated with pathological conditions, including T1D [9]. On the other hand, an impaired glucose tolerance has been reported in HDC^−/−^ mice [10], together with autoantibodies reactive to glutamic acid decarboxylase (GAD).

Histamine exerts important functions in allergy, inflammation, neurotransmission, and the gastrointestinal tract through G-protein-coupled specific histamine receptors, termed H1R-H4R. In the context of diabetic physiopathology, it plays a pivotal role in various physiological functions, such as feeding behaviour and energy homeostasis. Intracerebroventricular administration of histamine consistently decreases appetite in several species [11]. Mice with genes disrupted for HDC are prone to become obese on a high-fat diet or at advanced age. These metabolic changes presumably are due to the impaired regulatory loop involving hypothalamic histamine and H1R [12, 13]. H3Rs located on histamine neurons negatively regulate the synthesis and release of histamine. Consequently, treatment with imetit, a H3R agonist, reduced plasma histamine, leptin, and insulin levels and has anorexigenic effects in diet-induced obesity [14].

Histamine has been shown to regulate T cells by enhancing Th1-type responses through the H1R and downregulating both the Th1 and Th2 type responses through the H2R [15, 16]. Furthermore, the monoamine is believed to exert immune regulatory functions in myeloid cells [17]. It has recently been described that anti-inflammatory and immunomodulatory functions of histamine are mediated through the H4R. Thus, they can be reproduced using the H4R agonist clobenpropit, which has been shown to suppress IL-12 production, modulate dendritic cell migration [18, 19], and regulate the growth of cancer cells through H4R [20].

Autoimmune diabetes in NOD mice is characterized by a progressive mononuclear cell infiltration in the pancreatic islets of NOD mice and leads to beta cell destruction and hyperglycemia. Pancreas-infiltrating CD4^+^ and CD8^+^ T cells have a Th1 phenotype [21]. It has been recently demonstrated that leptin can reverse diabetes [22]. Assuming that histamine might be involved in the pathogenesis of diabetes, we investigated the relationship between endogenous histamine production and disease development in the NOD mouse model, using histamine-deficient HDC^−/−^ mice backcrossed on a NOD background. Here, we show that the lack of endogenous histamine provides a notable protection against T1D. We observed an increase of Ly6G^+^CD11b^+^ immature myeloid cells in these mice that was not correlated with diabetes onset, while IL-12 and IFN-*γ* levels in the serum were decreased, suggesting that endogenous histamine modulates the inflammatory pattern. Paradoxically, we found that T1D onset was also delayed when exogenous histamine was given to NOD. Our study supports a metabolic role of histamine during the pathogenesis of autoimmune diabetes in accordance with previous findings supporting its immunomodulatory functions.

## 2. Materials and Methods

### 2.1. Mice

Conventional NOD mice (K^d^, I-A^g7^, and D^b^) were bred in our animal facility at the Hôpital Necker. HDC^−/−^ mice generated by Ohtsu et al. [23] received a histamine-low diet (SAFE (Scientific Animal Food and Engineering)) to avoid exogenous uptake. We generated HDC-deficient mice by speed backcrossing the HDC-deficient allele against the NOD background. In order to confirm the allele maps of NOD diabetes susceptibility genes, we analysed several representative microsatellite markers: we chose the alleles related to MHC and numerous insulin-dependent diabetes genes. The microsatellite analysis confirmed that congenic mice retained the NOD diabetes susceptibility alleles tested. Then, mice were intercrossed to generate the HDC^−/−^ mice used in this study.

### 2.2. Reagents and Treatments

We purchased histamine and 4-methylhistamine (4-MH) from Sigma-Aldrich and clobenpropit dihydrobromide (CB) from Tocris. Histamine (4 mg/kg) was injected i.p. in 100 *μ*L of saline once a week for 10 weeks starting at 4 weeks of age. H4R agonists, CB (10 *μ*g/kg), 4MH (10 *μ*g/kg), and vehicle (control group for H4R-agonist) were injected i.p. in 100 *μ*L of saline once a week for 10 weeks.

### 2.3. Mediators and Histamine Assays

Leptin (Millipore), IFN-*γ* (R&D), IL-6 (R&D), and IL-12 (R&D) were quantified in sera from mice, using ELISA kits according to the manufacturer's instructions. Histamine was quantified by an automated continuous flow spectrofluorometric technique [24].

### 2.4. Monitoring for Autoimmune Diabetes and Treatment of NOD Mice

We treated NOD mice with histamine, CB, or 4-MH and monitored weekly for clinical signs of diabetes using Gluko-Test reagent sticks, to detect glucose in urine samples (Boehringer Mannheim, Meylan, France). When needed, glycemia was also measured in a drop of blood collected from the tail vein using a Reflolux S glucometer (Boehringer Mannheim). Incidence of diabetes was defined based on the discovery, upon serial monitoring, of glycosuria and hyperglycemia (fasting glycemia >2.5 g/L).

### 2.5. Adoptive Transfer

To induce T1D by adoptive transfer, purified CD4^+^ diabetogenic T cells from prediabetic NOD mice were injected i.v. into eight-week-old NOD RAG^−/−^ recipient mice with a CD11b^+^ population (5 × 10^6^ cells/mice) magnetically sorted from the bone marrow of HDC^+/+^ or HDC^−/−^ mice. Bone marrow cells were stained with CD11b^+^ MicroBeads (Miltenyi Biotec) then sorted by positive magnetic separation on LS columns (MACS Separator, Miltenyi). The incidence of type-1 diabetes was monitored as described above. A high incidence of T1D is elicited within 10 weeks after T cell transfer.

### 2.6. Flow Cytometry Analysis

Bone marrow (BM), spleen, and peripheral whole blood cells were recovered. BM and spleen cells were prepared as previously reported [25, 26] and adjusted to a final concentration of 1 × 10^6^ per mL in culture medium (PBS from GIBCO BRL). Cell suspensions were stained with the following appropriately labeled antibodies: CD11b, Ly6G- Ly6C, CD4, CD25, Foxp3, mPDCA-1, CD11c, CD115, MHC-II, CD80, and CD86 (BD Biosciences, Pont-de-Claix, France). Samples were acquired on a FACSCanto II cytometer (BD Biosciences). Mononuclear cells were gated according to forward- and side-scatter properties and analysed using the FACS Diva Software.

### 2.7. Statistical Analysis

Diabetes incidence was plotted using the Kaplan-Meier method, that is, nonparametric cumulative survival plot. Statistical comparison between curves was performed using the log rank (Mantel-Cox) test that provided the corresponding *χ* ^2^ values. When needed, statistical comparison of mean values was performed using Student's *t* test. Differences were considered significant when *P* < 0.05 (∗).

## 3. Results

### 3.1. Histamine-Deficient Mice Are Partially Protected against Type-1 Diabetes

**Figure 1:**
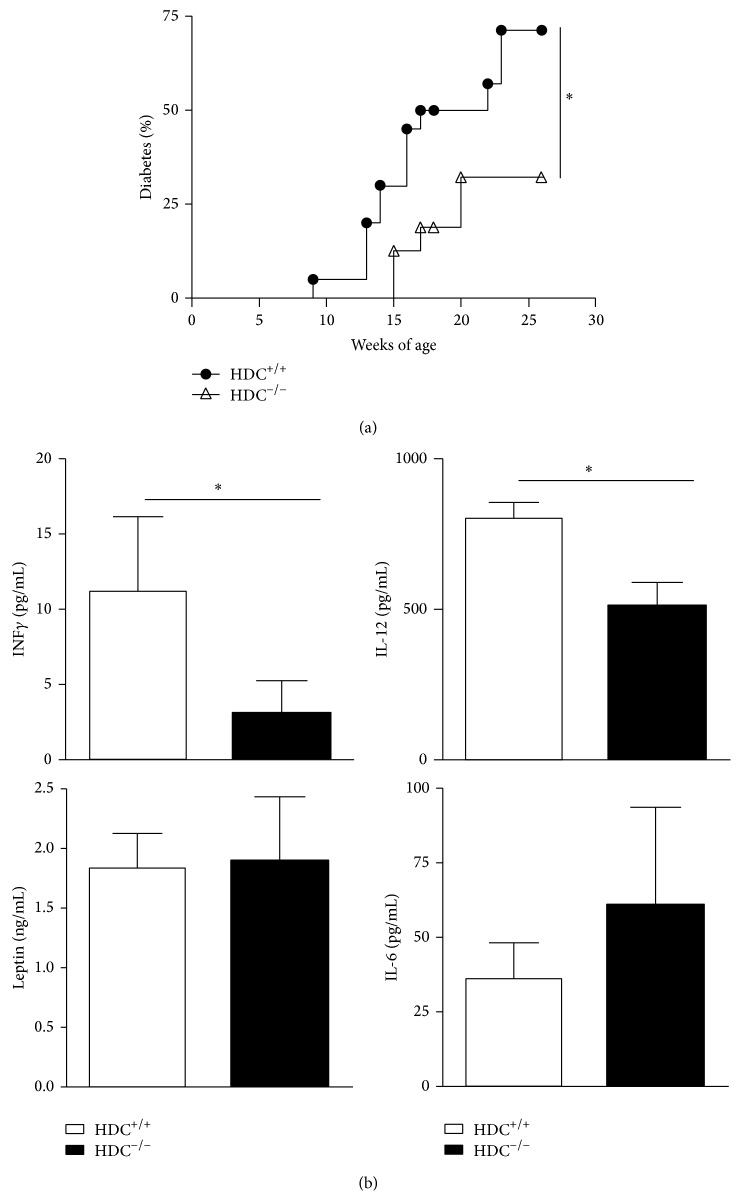
HDC^−/−^ mice are partially resistant to the development of autoimmune diabetes. (a) Cumulative incidence of spontaneous diabetes in female HDC^+/+^ (*n* = 22) and HDC^−/−^ strains (*n* = 20). Mice were considered diabetic when glycemia >2.5 g/L. (b) Serum levels of IL-6, IFN*γ*, IL-12, and leptin in HDC^−/−^ NOD and NOD female mice. For measuring leptin, serum was obtained from starved mice. Data are presented as means ± s.e.m. ^∗^ *P* < 0.05, HDC^−/−^ versus HDC^+/+^ mice determined by Student's *t*-test. (*n*  =  8).

To evaluate the role of histamine in autoimmune diabetes, we generated NOD mice deficient for the gene encoding HDC in the NOD genetic background. These genetically engineered mice were healthy and heavier than their wild-type controls after several months. As shown in Figure 1, the onset of T1D was delayed in NOD HDC^−/−^ mice and the disease incidence remained lower than that among wild-type controls, suggesting a deleterious role of histamine during pathogenesis. It may be hypothesized that an increase in plasma and cellular histamine content contributes to disease progression by enhancing endothelial permeability, as suggested for the intimal damage in atherosclerosis [8]. In favour of this hypothesis, histamine levels were effectively increased in diabetic NOD mice (data not shown), similarly to what has been reported for streptozotocin-diabetic rats.

### 3.2. Histamine Levels Affect Circulating IL-12 and IFN-*γ*, but Not IL-6 or Leptin

To assess potential changes in immunological and hormonal parameters in the absence of histamine, we analysed interleukin-12 (IL-12), IL-6, and leptin. IL-12 and IFN-*γ* levels were lower in HDC^−/−^ than in NOD mice, but no differences were noted for IL-6 or leptin (Figure 1(b)).

### 3.3. The Differentiation of CD11b^+^Gr-1^+^ Immature Myeloid Cells (IMCs) in HDC^−/−^ Mice Is Not Correlated with the Incidence of Diabetes

**Figure 2:**
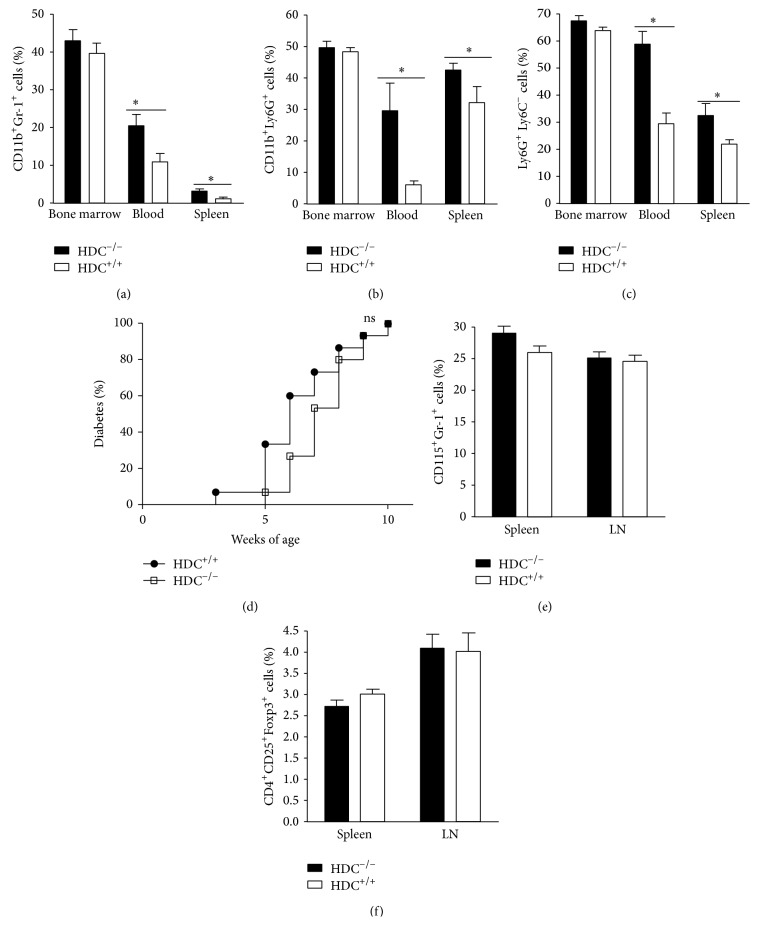
HDC deficiency increases CD11b^+^Gr-1^+^ and CD11b^+^Ly6G^+^ IMCs. (a) The percentages of CD11b^+^Gr-1^+^ IMCs in BM, spleen, and peripheral blood in wild-type and HDC^−/−^ mice were measured by FACS analysis (^∗^ *P* < 0.05; mean ± s.d. *n* = 5 for each group). (b) The relative proportion of CD11b^+^Ly6G^+^ cells in bone marrow, peripheral blood and spleen from wild-type and HDC^−/−^ mice measured by FACS analysis (^∗^ *P* < 0.05; mean ± s.d. *n* = 5 for each group). (c) The relative proportion of Ly6G^+^ Ly6C^−^ cells in bone marrow, peripheral blood and spleen of wild-type and HDC^−/−^ mice measured by FACS analysis (^∗^ *P* < 0.05; mean ± s.d. *n* = 5, each group). (d) Bone marrow-derived IMCs from HDC^−/−^ do not accelerate diabetes onset. Incidence diabetes was measured in NOD RAG^−/−^ mice after adoptive transfer of pathogenic lymphocytes and CD11b^+^ BM-derived IMCs from wild-type mice or HDC^−/−^ mice (*n* = 15, ^∗^ *P* < 0.05 compared to indicated control). (e) The percentages of Gr-1^+^CD115^+^ MDSCs in spleen and pancreatic LNs in wild-type and HDC^−/−^ mice were measured by FACS analysis (^∗^ *P* < 0.05; mean ± s.d. *n* = 5 for each group). (f) Percentages of CD4^+^CD25^+^ Foxp3^+^ regulatory T cells in spleen and pancreatic LNs in wild-type and HDC^−/−^ mice were measured by FACS analysis (^∗^ *P* < 0.05; mean ± s.d. *n* = 5 for each group).

As histamine contributes to the differentiation and maturation of hematopoietic myeloid cells [27], we performed a comparative cytometry analysis of these populations in histamine-deficient and wild-type NOD mice in various organs, such as bone marrow, spleen, and peripheral blood based on Gr-1, CD11b, Ly6G, and Ly6C expression by leukocytes. We found an increased percentage of immature CD11b^+^Gr-1^+^ cells in the peripheral blood and the spleen from NOD HDC^−/−^ mice (Figure 2(a)). A similar result was observed for the CD11b^+^Ly6G^+^ Ly6C^−^ subset from peripheral blood and spleen of NOD HDC^−/−^ mice (Figures 2(b) and 2(c)). These results confirmed the previously reported importance of histamine for the differentiation of CD11b^+^Gr-1^+^ myeloid subsets in a different genetic background. We hypothesized that the increased proportion of immature myeloid cells (IMCs) might be involved in the protection against type-1 diabetes in histamine-deficient mice. To test this assumption, we performed adoptive transfers of effector CD4^+^CD62L T cells together with CD11b^+^ bone marrow cells from HDC-deficient or WT mice to NOD RAG^−/−^ recipients, which did not alter diabetes onset (Figure 2(d)), ruling out the increase of IMCs as a cause for diabetes progression. We also analysed the status of myeloid-derived suppressive cells (MDSCs), which can modulate diabetes onset [28]. However, no difference was observed when comparing this subset in both strains (Figure 2(e)). Similarly, the proportion of CD4^+^CD25^+^Foxp3^+^ regulatory T cells remained unchanged (Figure 2(f)).

### 3.4. Exogenous Histamine Protects against T1D

**Figure 3:**
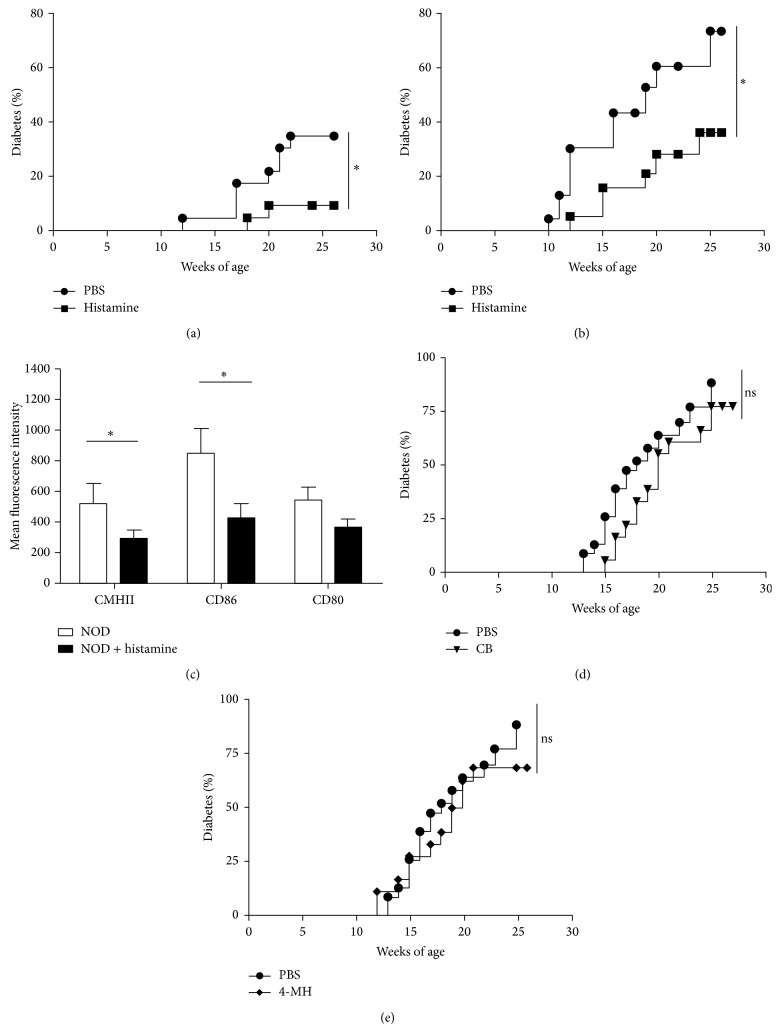
Effects of histamine, CB, and 4-MH on diabetes onset in NOD mice. (a) Effects of histamine deficiency in HDC^−/−^ mice on diabetes onset versus WT controls. Diabetes prevention in NOD HDC^−/−^ mice treated with histamine (4 mg/kg, i.p. injection, once a week for 10 weeks) (^∗^ *P* < 0.05; *n* = 18, each group). Mice were measured as diabetic when glycemia >2.5 g/L. (b) Similar experiment performed with NOD HDC^+/+^ mice (^∗^ *P* < 0.05; *n* = 18, each group). (c) Mean fluorescnce intensity of MHCII, CD80, and CD86 molecules on mPDCA1^+^CD11c^low^ pDCs (^∗^ *P* < 0.05; mean ± s.d., *n* = 5 per group). Effects of CB (d) and 4-MH (e) on diabetes onset in WT mice (*n* = 18, each group).

Since the generation of endogenous histamine appears to enhance the pathogenesis of T1D, we attempted to reproduce this deleterious effect by adding exogenous histamine. Wild-type and NOD HDC^−/−^ mice received i.p. injections of either vehicle (100 *μ*L PBS) or histamine (4 mg/kg), respectively, once a week for 10 weeks. As shown in Figures 3(a) and 3(b), exogenous histamine not only failed to increase the incidence of T1D, but also delayed the onset of disease in both wild-type and HDC^−/−^ mice. We further analysed whether histamine treatment modulated the expression of costimulatory molecules on DCs. As shown in Figure 3(c), decreased expression of class II major histocompatibility complex (MHC-II) and CD86 molecules occurred only on pDCs from pancreatic lymph nodes (LNs) in mice treated with histamine, suggesting that this mediator may modulate the activation pattern of APCs.

### 3.5. H4R Agonists Fail to Induce Protection against T1D

Recent evidence supports the notion that the inflammatory functions of histamine are mediated through the H4R [20, 29, 30]. For this reason, we examined whether compounds characterized as potent H4R agonists could reproduce the protective effect provided by exogenous histamine. We injected NOD mice with clobenpropit (CB), an isothiourea derivative of histamine, or 4MH for 10 weeks and found no significant protection as compared with mice having received vehicle alone (Figures 3(d) and 3(e)).

## 4. Discussion

In the present study, we provide evidence that histamine deficiency causes a delay in diabetes onset and decreases its incidence. This result fits with clinical observations in diabetic patients since increased histamine levels were associated with pathogenesis in these patients [3]. Furthermore, there is now a compelling body of evidence for the contribution of histamine to the development of autoimmune diseases from animal studies [31]. It has also been reported that inhibition of histamine synthesis in experimental diabetes reduces disease complication [32]. Several arguments suggest that in the present study the phenotype results from vascular and/or metabolic functions of histamine. This conclusion has already been proposed in a rat diabetic model, since the authors have shown that the gastric histidine decarboxylase activity and plasma gastrin levels were increased in connection with the depletion of insulin [9]. Another argument derives from the observation that histamine deficiency decreases atherosclerosis and inflammatory responses [8]. Similar to our model, lowering the level of endogenous histamine was protective. We can thus anticipate that decreasing histamine levels in diabetic patients can decrease the risk of vascular complications, which is one critical issue in the cure for patients.

There is increasing evidence that diabetes is under the control of numerous cytokines and hormones. Since leptin can influence diabetes [22, 33], we have measured the level of this mediator in serum of starved mice of both strains. However, we observed no change in its level. We observed that IL-12 and IFN-*γ* levels were lower in NOD HDC^−/−^ female mice compared with WT controls. These cytokines are known for promoting diabetes progression in the NOD strain, possibly via induction of Th1-type cytokines [34, 35]. By contrast, circulating IL-6 or leptin did not differ between WT and KO mice, ruling out a major involvement of these factors in the delayed disease onset in HDC-deficient mice.

Even though we detected hematopoietic anomalies in the myeloid population of HDC^−/−^ mice, these cells were not involved in the protective response, as demonstrated by adoptive transfer. Although neutrophils express the same markers as IMCs, the protection against diabetes in HDC-deficient mice cannot be ascribed to an increase of this population among the CD11b^+^Gr-1^+^ subset since neutrophil depletion has been shown to inhibit type-1 diabetes development in NOD mice [36]. Furthermore, the expansion of IMCs is not concomitant with MDSCs since the two populations bear different markers and most MDSCs do not express Ly6G. Furthermore, we found that the proportion of regulatory myeloid cells was not modified comparing both strains. Finally, the increased histamine levels associated with the pathogenesis of diabetes are expected to be a consequence rather than a cause of disease. Therefore, adding exogenous histamine may act on the gastric mucosa to enhance the flow of the lipidic factors but will be unable to reverse the phenotype observed in HDC-deficient mice. Indeed, we observed the opposite effect.

We describe a new immunomodulatory function of histamine in our model since diabetes onset is delayed in both wild-type and HDC^−/−^ mice after treatment. It is plausible that the protective effect is mediated through several histamine receptors. For example, the role of the H3R on food intake has been well documented. Since it has been previously established that H4R drives inflammatory responses in asthma, dermatitis, arthritis, and colitis [29, 30, 37], we hypothesized that its agonists could reproduce the effects of histamine. This was not the case in this study, but even though we could not validate the H4R pathway using the agonists CB or 4-MH, we cannot exclude that the dose, the route of administration, or the choice of the agonists tested was inadequate. Alternatively, a mixture of H2R and H4R agonists may be required to obtain a protective effect similar to the one induced by histamine.

Histamine, which is increased in inflammatory and pathological situations, has also been shown to decrease, in vitro, the production of IL-12p70 of monocyte-derived dendritic cells (DCs) via H2R and H4R [17, 19, 38] and to inhibit IL-27 production in activated APCs [39]. The regulation of cytokine production in maturing DCs causes an alteration of the T cell polarization that has been well described [15, 40, 41]. More recently, it has been reported that histamine decreases the migration of human pDCs and their cytokine production, while increasing regulatory T cell recruitment [42]. In histamine-treated NOD mice, we observed that pDCs from pancreatic lymph nodes expressed lower levels of costimulatory and MHC-II and CD86 molecules than their counterpart from control mice, consistent with a decreased inflammatory state. However, the exact mechanisms underlying histamine-mediated protection deserve further investigation.

Contrary to our anticipations, injection of histamine did not promote diabetes pathogenesis but, paradoxically, decreased disease onset. The immunomodulatory function of histamine has been described in other inflammatory models [43] and histamine is used therapeutically in patients suffering from acute myeloid leukemia [44]. Histamine release can be induced by different stimuli, such as cytokines or microbial stimulation. It has been previously shown that human basophils release histamine in response to a Toll-like receptor 2 (TLR2) agonist [45]. Interestingly, TLR2 agonists have also been implicated in the modulation of T1D in the NOD mouse [46]. Similarly, parasites such as* Schistosoma mansoni* protect NOD mice [47] and parasites stimulate basophils and induce histamine release [48]. These results support our hypothesis that local histamine release upon stimulation of immune cells, such as basophils, may participate in immunoregulatory pathways in autoimmune diabetes.
